# Hydrogel injection outside of prostate radiation: an overview

**DOI:** 10.1007/s00261-025-05288-x

**Published:** 2025-11-13

**Authors:** Stephen Polanski, J. Louis Hinshaw, Chris Gu

**Affiliations:** 1 University of Wisconsin–Madison, Madison, USA; 2 Mayo Clinic, Rochester, USA

**Keywords:** Hydrogel injection, Hydrogel, SpaceOAR, Cross-sectional Intervention, Extraprostatic, Injection

## Abstract

As radiotherapy has evolved and improved, it has become possible to apply higher doses of radiation more safely. However, adjacent structures and the associated collateral damage remains an important consideration. Creating separation between a target organ and these organs at risk (OAR) is fundamental to allowing greater and potentially more effective radiation coverage using external beam radiation. This technique has initially been described in the setting of prostate cancer with hydrogel being injected to separate the prostate from the rectum. The purpose of this manuscript is to describe both prostate and non-prostate techniques for utilizing hydrogel for OAR displacement in the setting of high dose radiation therapy with the intention of increasing target dose while limiting off target radiation to safe levels.

## Background

Learning Objectives:

1. Identify indications and patient selection.
2. Describe optimal injection techniques.
3. Describe potential complications.

Cancer remains one of the leading causes of death globally. The International Agency for Research on Cancer estimates 9.7 million cancer deaths worldwide with an incidence of nearly 20 million. (GCO IARC website). While there are a variety of potential treatment modalities available, radiation therapy is a relatively common treatment modality with nearly 50% of all cancer patients receiving radiation as a form of therapy during the course of their treatment [1]. In adequate doses, radiation results in cellular death with a multifactorial mechanism of action that is dose dependent and complex. Applying adequate tissue dose to a target tumor while sparing adjacent tissues is a process that has undergone significant advancement over time in the last several decades, particularly with the development of stereotactic body radiation therapy (SBRT). SBRT is the application of radiation from multiple projections focused on a specific anatomic location, allowing higher doses to the target tissue with relatively low doses to nontarget tissues traversed on the way to the target. This allows dose related tissue ablation at the level of the target tumor rather than relying on the relative radiosensitivity between the tumor and nearby normal tissues as is seen in conventional fractionated radiotherapy [2]. Applying the radiation from multiple projections does decrease the dose to intervening non-target tissues, but if radiation sensitive tissues are immediately adjacent to the target tumor, there can still be significant and dose limiting associated toxicities. Thus, the need for a method to physically displace these “organs at risk” (OAR) from the target tumor prior to therapy such that these toxicities are limited but the target tumor receives a high enough dose to ensure a high likelihood of complete tumor necrosis. Since these treatments occur over the course of weeks to months, the displacement needs to persist over at least that time period. This has led to the development of hydrogels for this purpose with initial FDA approval in the setting of rectal displacement for prostate cancer radiation.

The goal of radiation treatment is to cover 95% of the target volume with 95% of the prescribed dose. When assessing tumor dosing, the percentage of the tumor volume receiving 95% of prescribed dose is denoted as planning target volume (PTV). If the PTV is less than 95% due to anatomic limitations related to off target tissue toxicity, then the treatment will generally be suboptimal with a higher likelihood of local failure. In the prostate, utilizing a hydrogel to displace the adjacent rectum has been associated with rectal displacement of 1 cm and more with a mean increase of 1.26 cm of perirectal spacing. In clinical trials, this has resulted in a significant decrease in rectal dose despite much higher prostate dosing [3, 4]. These promising results have led clinicians to apply this technique routinely in clinical practice. Utilizing this technique in other anatomic locations, while hypothetically advantageous, has not been widely accepted or studied. However, it is an important potential clinical tool and delineating associated techniques to apply it safely in these settings should be explored. The purpose of this manuscript is to describe the principles of image guided hydrogel injection in both periprostatic and other non-standard anatomic locations with a focus on patient selection, image guidance, technical considerations, and potential complications.

## Periprostatic utilization

The use of hydrogel has been well established as a safe and effective way of limiting radiation toxicity on the rectum during radiotherapy treatment of prostate cancer [5]. This FDA approved procedure involves injecting an absorbable hydrogel spacer in the perirectal space between the prostate and anterior rectal wall, which temporarily displaces the rectum away from the prostate and thus reduces the radiation dose to the rectum during radiotherapy [6]. Along with decreased rectal radiation dose, meta-analyses have shown that perirectal hydrogel spacer placement is associated with decreased gastrointestinal and genitourinary toxicities and higher bowel-related quality of life [7]. While this procedure is often performed by urologists and radiation oncologists, it is also performed by radiologists at some institutions.

**Fig. 1:**
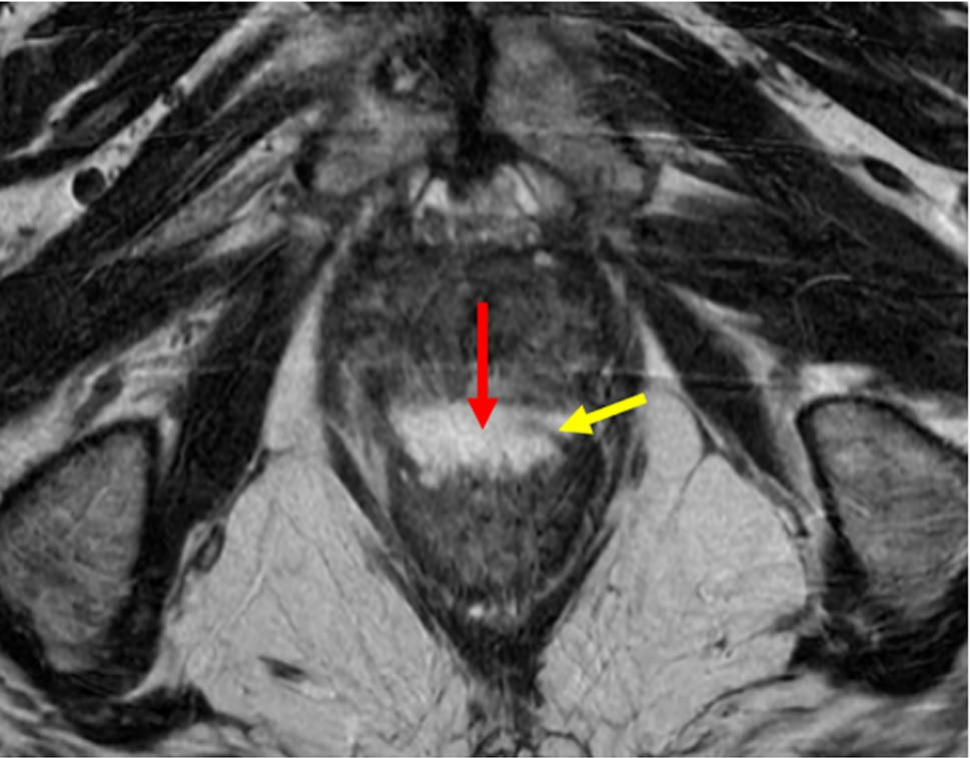
Routine post procedural axial T2 MRI shows infiltration of hydrogel material (red arrow) into the anterior rectal wall (yellow arrow) without disruption of the mucosa. Patient was asymptomatic during and after the procedure

Complications from hydrogel spacer placement are typically mild and self-limiting, with the most common being mild post procedure discomfort [8]. Serious complications from hydrogel spacer placement have been reported, although without event rates, and include rectal or urethral injuries [9]. While rectal wall infiltration of hydrogel material is a finding that can be seen on routine post procedural MRI [10], the rate of occurrence is not well known. In our experience, this is an asymptomatic finding that rarely needs treatment (prophylactic antibiotics) (Fig. 1). Antibiotic recommendations are specific to the institution but should cover common enteric and urinary pathogens.

## Off label anatomic utilization

Patient selection is important in achieving the optimal results for SpaceOAR injection. With limited overall experience injecting spacer hydrogel in various anatomic locations, certain assumptions have been made based upon our experience with hydrodissection in the setting of thermal ablation. This is a technique that has been well described in the literature, involving injecting a comparatively large volume of fluid intended to temporarily displace nontarget tissues to protect them from thermal damage during image guided thermal ablation. While the medium is different, the goal is the same, and lessons learned in that arena can be expected to carry over to this indication as well. The anatomy between a tumor being targeted for radiation, and the adjacent OAR should be assessed for potential spaces where the hydrogel can be injected, previous interventions that could lead to scarring and fibrosis that may limit displacement, and the possibility of complications related to migration of the hydrogel. Considering the relatively low volume of hydrogel associated with each injection, there should be realistic expectations around the amount of displacement that can be achieved, both as it relates to how far the OAR can be displaced from the target tumor (~ 1 cm) and the total expanse of contact that can be addressed (i.e. the surface area of contact between the OAR and the tumor). Based upon experience with the prostate, it would be expected that a surface area of approximately 6–12 cm^2^ could be addressed with a single injection, although this needs more study.

As with all image-guided percutaneous biopsies, bleeding risk and anticoagulant use should be considered. While hydrogel injection would be defined as a low risk procedure, visceral injections are inherently associated with some degree of risk for a hemorrhagic complication. Based upon most recent SIR guidelines, as a low-risk procedure, PT/INR and platelets do not need to be routinely ordered prior to the procedure unless there is an indication to do so (Patel). Although no specific cut off values are known for this procedure, an INR < 2 and platelet count > 25,000 would be a reasonable consideration if those parameters are assessed.

Medications should be reviewed prior to the procedure. While it is a low-risk procedure, medications that inhibit platelet function or otherwise interfere with normal coagulation should be held according to institutional guidelines for low-risk percutaneous procedures. For example, at our institution, ADP inhibitors are held for 5 days and Phosphodiesterase inhibitors are held for 3 days prior. Full recommendations are followed based on Mayo Clinic guidelines for interventional radiology anticoagulation [11].

**Fig. 2:**
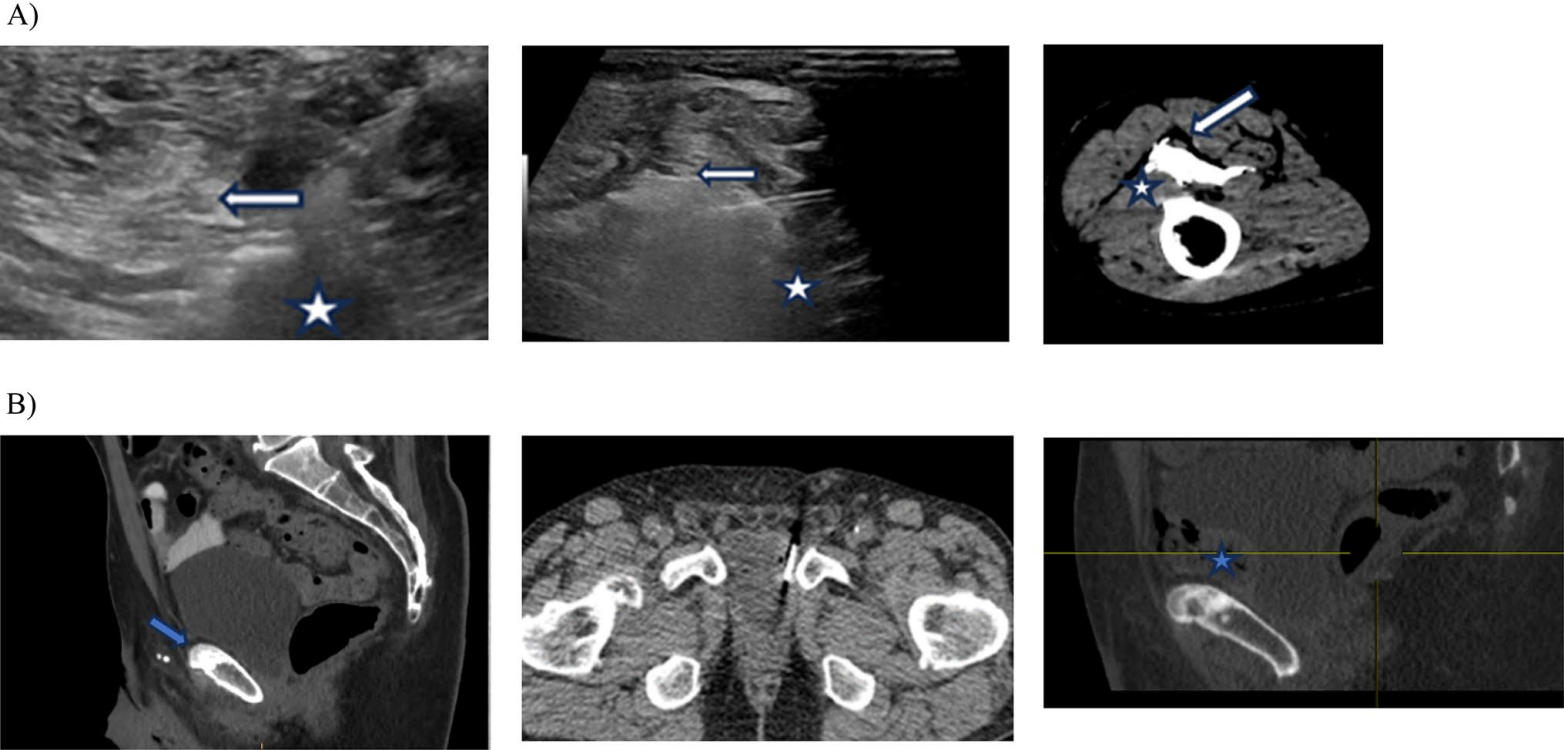
**A** Ultrasound showing a metastatic lymph node (white star) in the popliteal fossa and the adjacent neurovascular bundle (white arrow). Real time ultrasound image showing the hydrogel injection which creates a snowstorm appearance. The neurovascular bundle is partially obscured by the gel. Post procedure confirmatory CT demonstrating adequate displacement between the lymph node and the neurovascular bundle. **B** CT image of a sclerotic osseus metastasis in the pubic symphysis (blue arrow) abutting the bladder. SpaceOAR injection using CT guidance as US was limited due to osseous structures. Post procedure CT reconstruction demonstrating SpaceOAR hydrogel (blue star) displacing the bladder away from the osseous metastasis

When performing these procedures the patient should be positioned as needed to optimize image-guided positioning of an appropriate needle into the anatomic space between the target tumor and the OAR, preferably without traversing any solid organs or bowel. Image guidance can be performed with any standard guidance modality that allows accurate needle placement and imaging of the pertinent anatomic structures with CT and ultrasound being by far the most common. The decision between CT and ultrasound should be based on each individual case, the provider’s comfort level with those modalities, and patient related factors just like any other image-guided procedure. In addition to the standard advantages of decreased cost, lack of radiation, and time efficiency, ultrasound has the additional advantage in this setting of allowing real time monitoring of both test saline injection and hydrogel injection, increasing the likelihood that the injection will occur into the targeted anatomic space, similar to when the procedure is performed in the standard periprostatic space (Fig. 2A). Thus, US should be favored when possible. However, in some scenarios, CT does have advantages, including: visualization of certain structures that can be difficult to identify with US (Fig. 2B); precise visualization of needle placement in deeper anatomic locations; and direct correlation of needle placement with prior cross sectional imaging, thus ensuring placement of the hydrogel at the precise location needed for tissue displacement. With modern CT fluoroscopy techniques, mm level accuracy of needle placement is possible, which is generally necessary since for this procedure to be indicated, the target tumor and OAR will by definition be often be within 1–2 mm of each other.

## Targeting and preparation

Preprocedure assessment should start with review of all relevant imaging and patient suitability prior to the procedure. At this time, one should determine if a needle can be safely positioned in the potential space between the tumor and the OAR, if the surface area of apposition is appropriate, what guidance modality will be most appropriate, and a plan for a safe planned approach. One should also confirm that the patient will be able to tolerate the procedure based upon associated comorbidities and any limitations. Similar to other image-guided procedures, local anesthesia is generally adequate for more superficial targets, but conscious sedation may be more appropriate for any deeper targets. Standard precautions should be followed for moderate sedation as per your institution’s policies. Final preparation includes discussion of indications, risks and expectations with the patient prior to the procedure. Although current experience is not broad enough to provide specific expectations for complications and outcomes, it is likely that the complications will closely parallel other percutaneous needle based procedures and in our experience, the large majority have been successful in providing adequate displacement.

**Fig. 3:**
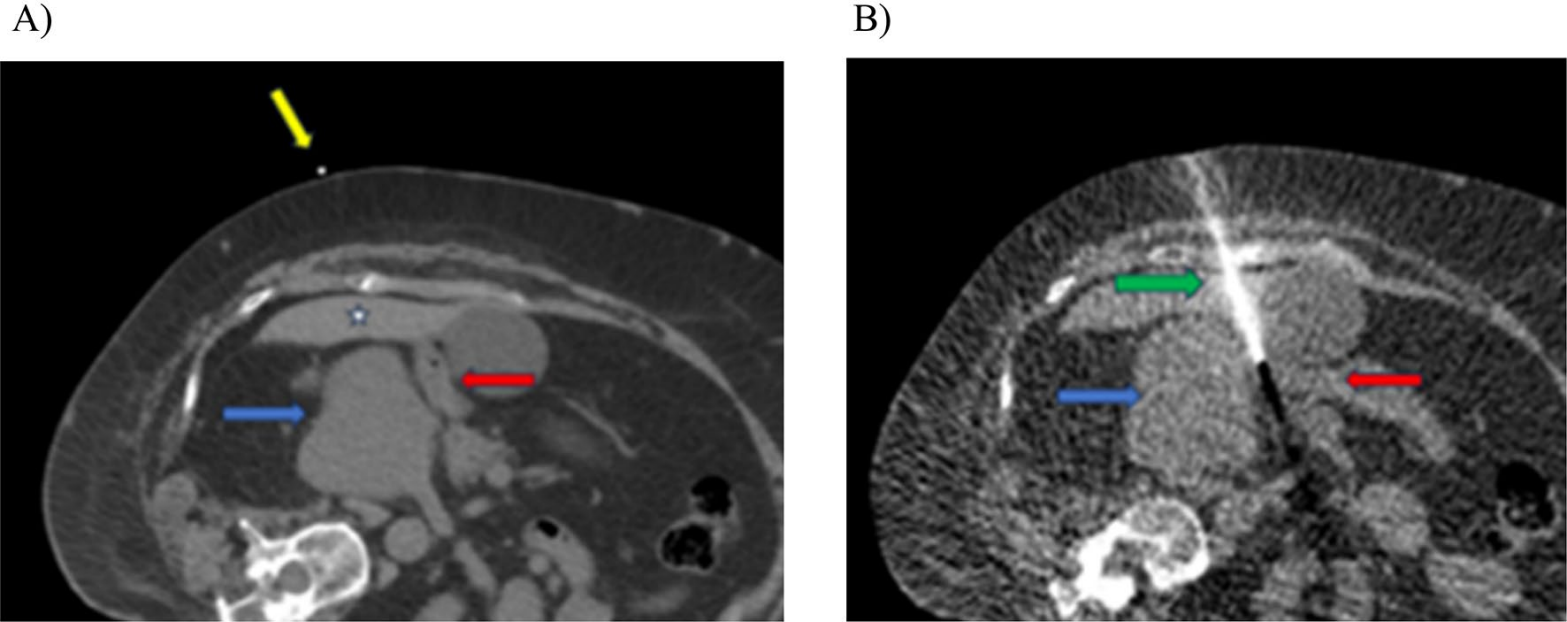
**A** Initial planning for SpaceOAR injection. Large retroperitoneal mass (blue arrow) abutting the adjacent small bowel (red arrow). Note a dilator external to the patient on initial scan (yellow arrow) helps with needle entry site. In this case traversal of the liver (star) was unavoidable for needle placement. **B** Needle in place (green arrow) with tip between the mass (blue arrow) and the bowel (red arrow)

For CT targeting the patient is placed on the table in the position that is preselected to be the best to access the target and standard CT guidance techniques are then utilized to plan and execute the safest and most appropriate needle placement. We prefer utilizing CT fluoroscopic guidance techniques due to the high precision associated with the technique, but any technique can be utilized as long as the needle placement is accurate. The needle should be positioned with the tip positioned in the approximate geographic center of potential space between the tumor and the OAR, preferably without traversing any solid organs or bowel although solid organ traversal may occasionally be required for optimum placement (Fig. 3). This allows for expansion of the potential space with the hydrogel generally extruding relatively symmetrically in all directions from the needle tip. Note that there is a slight preference for extruding further along the trajectory of the needle rather than perpendicular to the needle and that should be considered.

Ultrasound targeting is similarly performed much like any other US-guided procedure. The patient should be positioned comfortably in a way that allows the safest and easiest path to the target. After visualization of the target on grey scale, vessels should be assessed with color doppler prior to choosing an insertion angle. Our preference is to utilize a needle guide for increased precision, but again, any technique that allows accurate needle positioning is appropriate.

Standard sterile field technique is required for this procedure. Although the risk of associated infection is unclear, as with all procedures that involve the injection of a foreign substance that will remain in place, sterility is of paramount importance since an associated infection could be particularly problematic.

After preparation, standard local anesthesia should be utilized. We prefer an infiltration of the skin and subcutaneous tissues using 1% Lidocaine with epinephrine buffered with 8.4% sodium bicarbonate in a 9:1 ratio, but any appropriate local anesthetic will work. Once appropriate local anesthesia and any needed conscious sedation has been administered, then needle placement can be performed. Any needle 19 gauge or larger can be utilized. Note that 18 gauge is what is utilized for prostate injections and thus, if possible, is optimal for this technique. Anything smaller than 19 gauge is problematic due to the high viscosity of the spacer gel.

## Test injection

**Fig. 4:**
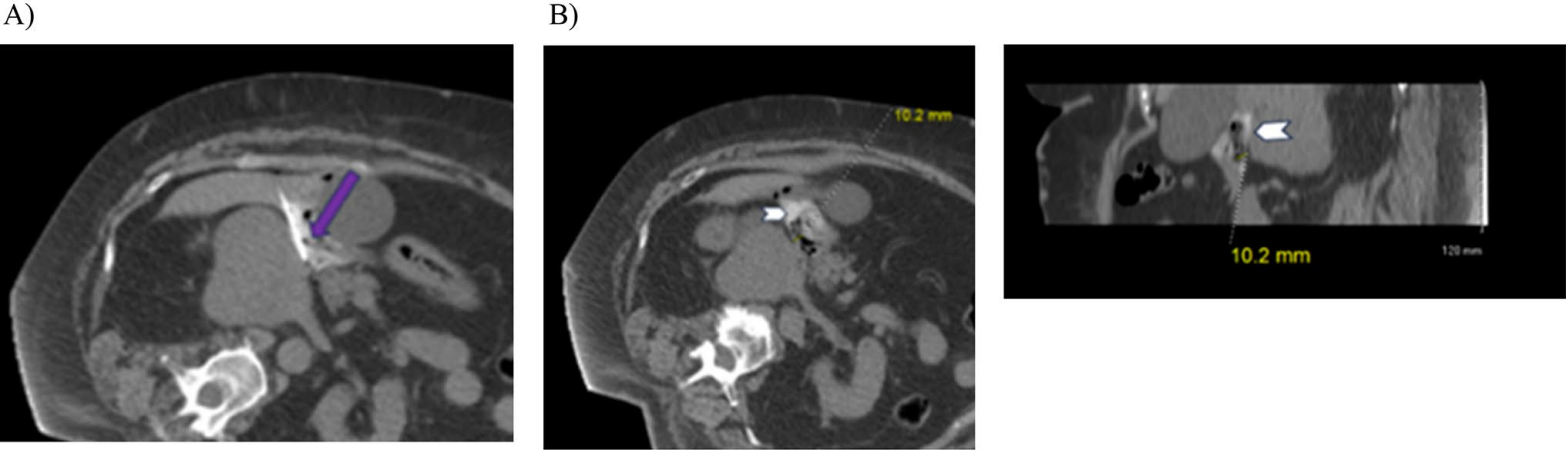
**A** Test injextion of iohexol doped saline (purple arrow). 1–2 ml injected and confirmed to be in the correct anatomic space, with slight displacement of the bowel. **B** 10 ml of SpaceOAR (arrowhead) was injected into the correct space after confirmation on test injection. Confirmation of appropriate displacement was done on limited volumetric scan with axial and sagittal reformats shown. Roughly 1 cm of displacement was achieved

Once the tip of the needle is appropriately positioned, a test injection should be performed (Fig. 4A). Although any fluid that can be visualized with the utilized guidance modality is appropriate, we have found that normal saline works well for ultrasound guided procedures and normal saline doped with 2% Iohexol as previously described for hydrodissection works well for CT guided procedures [12]. Generally, only 1–2 ml is necessary to confirm that the fluid is going into the appropriate anatomical location, but more can be utilized as necessary, particularly if repositioning is required. Following a successful test injection showing the fluid is filling the appropriate space, the hydrogel injection can be performed (Fig. 4B).

### Prostate hydrogel injection

Under ultrasound-guidance, SpaceOAR hydrogel is administered as follows. SpaceOAR hydrogel is prepared as described in the manufacturer’s “Instructions For Use”. Further details of the SpaceOAR kit are described in the next section. With the patient maintained in the dorsal lithotomy position, the transrectal ultrasound probe is positioned to enable direct ultrasound guidance of the needle into the space between the prostate and the rectum. Following appropriate sterile preparation of the perineum and appropriate utilization of lidocaine for local anesthesia, direct ultrasound guidance is employed via a transperineal approach to place an18G needle through the rectourethralis muscle into the perirectal fat inferior to the prostate. The needle position should be in the midline with the tip at the level of the mid gland, which should be confirmed in both sagittal and axial fields. A test injection used to confirm appropriate needle tip position between the prostate and anterior rectal wall (Denonvilliers’ fascia). If resistance is felt during the test injection or if the injected lidocaine is found to be in a suboptimal position, adjustments can be made to ensure the needle tip is in the appropriate position. The lidocaine syringe is then removed and the assembled SpaceOAR delivery system is attached to the 18G needle.

**Fig. 5:**
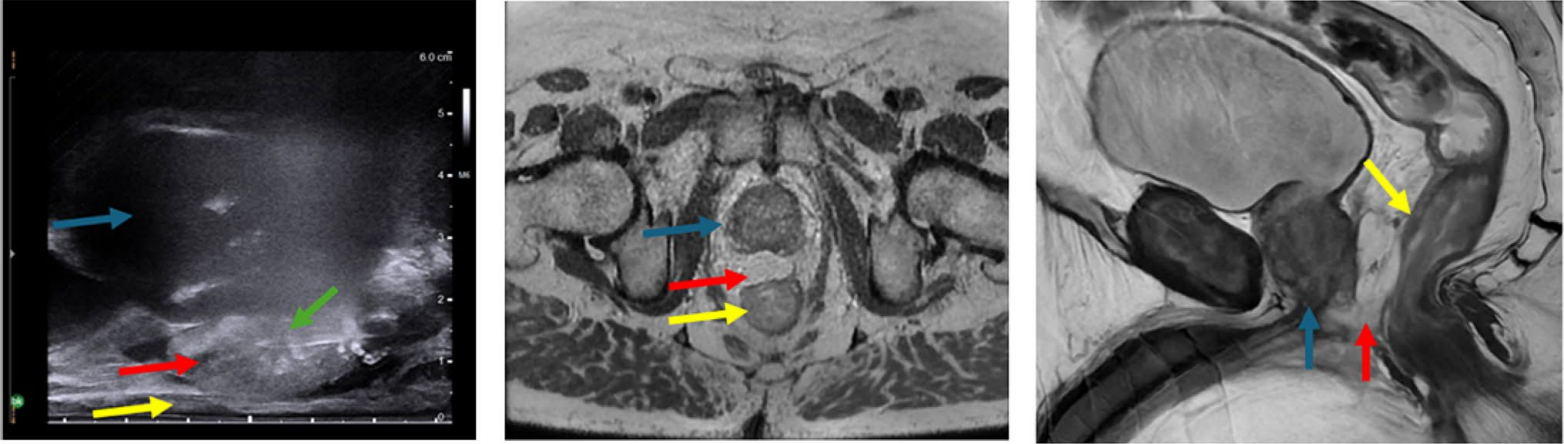
Longitudinal ultrasound image after injection of hydrogel spacer (red arrow) in the perirectal space between the prostate (blue arrow) and anterior rectal wall (yellow arrow) via an 18G needle (green arrow). Subsequent axial and sagittal T2 MRI demonstrates appropriate position of hydrogel spacer (red arrow) between the prostate (blue arrow) and rectum (yellow arrow)

Under ultrasound monitoring (sagittal plane), a smooth, continuous injection technique is used to dispense the SpaceOAR hydrogel into the space between the prostate and rectum (Denonvilliers’ fascia and the anterior rectal wall) (Fig. 5). The entire syringe contents (10 mL total) is injected without stopping. Optimal visualization of the needle during hydrogel administration is maintained at all times. Care is taken to ensure that penetration or compromise of the rectal wall does not occur. The needle is then removed and local pressure held until hemostasis is achieved.

## Extraprostatic injection

Although we have utilized SpaceOAR gel at our institution, there are other hydrogels available, which may be appropriate as well. The Space OAR kit is single use and is formed by mixing two solutions, the precursor and the accelerator. Specific instructions regarding preparation of the solutions is found in the package insert. The precursor solution is created with the Diluent solution (Trilysine buffer) with polyethylene glycol (PEG) powder. The accelerant is a salt buffer that cross links to create a soft hydrogel once mixed.

**Fig. 6:**
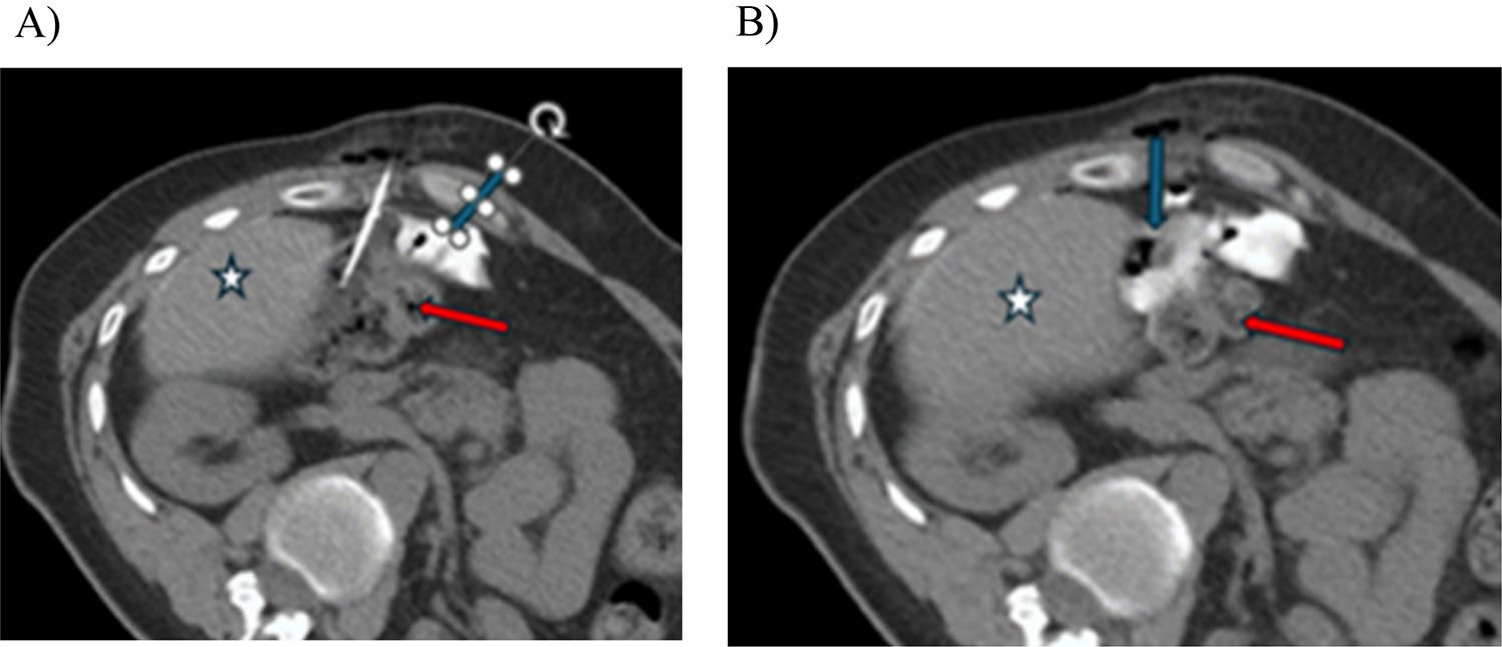
**A** Initial injection (blue arrow) between the liver mass (star) and adjacent bowel (red arrow) extruded into the space around the bowel with minimal displacement. **B** After appropriate needle repositioning, a subsequent injection (blue arrow) of an additional 10 ml of hydrogel achieved appropriate displacement between the bowel (red arrow) and adjacent liver mass (star)

After mixing the Diluent syringe with the PEG powder, the syringe is then connected to the provided Y connector. The Accelerator is also connected to the Y connector followed by placement of a syringe holder and plunger cap. The plunger cap allows for equal mixing of the two syringes. The kit should be used within an hour after opening. Once the injection has been started, the entire mixture should be injected in one smooth push. This is because if the injection is paused, the gel will solidify in the needle and the needle will become clogged, necessitating a second needle placement and new kit. Specifically, once the injection is started the gel will stabilize and solidify within roughly 10 s. The spacer gel should not be injected through intervening tubing prior to the coaxial needle but rather should be injected at the needle hub both to decrease wasted volume and to prevent solidification within the tubing. When US guidance is utilized, the injection can be monitored directly, but with CT guidance, it can only be evaluated after the injection has been completed. After the injection, the hydrogel placement should be confirmed with CT. If the desired goal of displacement is not obtained, an additional injection can be performed (Fig. 6). Multiple injections may be necessary for adequate displacement of the OAR, and desired displacement should be discussed with the radiation oncologist prior to procedure. There is no current documented upper limit to the number of injections that can be performed, and the hydrogel is considered biodegradable and inert. Noncontrast CT is sufficient as the hydrogel is iodinated and typically contains small amounts of gas, both of which help with visualization.

## Potential complications

Image guided percutaneous injections are frequent procedures within the United States. Risk of significant hemorrhage is very low in these patients with appropriate technique and attention to arterial anatomy, especially since these injections are not being performed into a solid organ, but rather in the space around the organs. This would be considered a low-risk procedure with a < 1.5% rate of major bleeding as defined by the society of interventional radiology [13]. Based upon experience with hydrogel injection in the periprostatic region, the rate of infection has been quoted at 3% in one study, although the rate of infection for all abdominal procedures has been cited using ultrasound guided interventions to be as low as 0.2%, so the higher rate associated with periprostatic injections may be related to the close proximity of the rectum and associated opportunity for contamination [14, 15]. Uncommonly, there can be intravascular injection of hydrogel or air; because of the more viscous nature of the hydrogel, it can act as an embolic agent when injected intravascularly with extremely rare cases of pulmonary embolism reported with prostate injection, 9 instances between 2015 and 2022 out of a total of 990 SpaceOAR related complications [16].

## Post-procedural care

Patient management after hydrogel injection includes appropriate treatment of any post-procedural pain, recovery from administered sedation, and 1–2 h of observation. To date, post-procedural pain has been self-limited and has resolved with appropriate pain medications. Other post-procedural care is as per standard recommendations after any percutaneous low risk procedure.

## Conclusion

Spacer hydrogel injection has been FDA approved to separate the rectum from the prostate prior to prostate cancer radiation, a technique that has been used successfully to decrease radiation to the rectum and increase target dose to the prostate cancer. However, there are many non-prostate cancer malignancies being targeted with radiotherapy that are closely opposed to OAR that can also potentially benefit from hydrogel injection. This manuscript describes some techniques for safely and effectively utilizing this procedure in these patients based upon our early clinical experience. In our institution, this has resulted in significant improvements of tumor radiation dosing and decreases off target damage without any significant complications. Appropriate patient selection and procedural planning are key to successful and safe injections. Overall, preprocedural planning and needle guidance techniques are essentially identical to other percutaneous trochar procedures and hydrogel injection is reminiscent of hydrodissection techniques utilized in the setting of percutaneous thermal ablation. The procedure has minimal risk, a significant potential benefit, and should be considered for any patient being considered for radiation treatment that has an associated dose limiting OAR.

## Data Availability

No datasets were generated or analysed during the current study.
